# Management of iron deficiency in women of childbearing age with oral iron intolerance: a prospective, randomised, controlled trial of three doses of an iron-whey-protein formulation: Prospective RandomisEd study of women of Childbearing age with gastroInteStinal Intolerance to Oral iroN (PRECISION)

**DOI:** 10.1007/s11096-023-01640-7

**Published:** 2023-12-26

**Authors:** Mark Ledwidge, Fiona Ryan, Anna Seoighe, Maria Jose Santos-Martinez, Cristin Ryan, J. G. F. Gilmer

**Affiliations:** 1 School of Pharmacy and Pharmaceutical Sciences, Trinity College Dublin, Dublin, Ireland; 2 Solvotrin Therapeutics, Little Island, Cork, Ireland; 3 School of Medicine, University College Dublin, Dublin, Ireland; 4 School of Medicine, Trinity College Dublin, Dublin, Ireland

**Keywords:** Adherence, Gastrointestinal intolerance, Iron deficiency, Women of childbearing age

## Abstract

**Background:**

Nutritional deficit and oral iron gastrointestinal intolerance may be a common cause of iron deficiency, which can be managed by pharmacists.

**Aim:**

To understand the prevalence of iron deficiency in women of childbearing age with a self-reported history of intolerance to oral iron and the tolerability of three doses of an iron-whey-protein formulation in the care of these women.

**Method:**

Ferritin and haemoglobin levels were documented in women of childbearing age with oral iron gastrointestinal intolerance. In those with iron deficiency (ferritin < 30 µg/L), adherence, gastrointestinal tolerability, ferritin, transferrin saturation and haemoglobin levels were compared between their prior oral iron product and iron-whey-protein microspheres randomised to three doses (14 mg daily, 25 mg daily and 50 mg daily) for 12 weeks.

**Results:**

Most screened women had low iron stores (128 (62.7%); ferritin < 30 µg/L), 65 (31.9%) had moderate to severe iron deficiency (ferritin < 12 µg/L) and 33 (16.2%) had iron deficiency anaemia (ferritin < 30 µg/L, haemoglobin < 12 g/dL). Amongst the 59 women who participated in the prospective clinical study of iron-whey-protein microspheres over 12 weeks, 48 (81.4%) were classified as adherent/persistent and fewer instances of gastrointestinal intolerance were reported (0.59 ± 0.91) when compared to 12 (20.3%) and (4.0 ± 2.2) respectively while taking the prior oral iron (Fisher’s Exact and* T*-test respectively, both *p* < 0.001). There was no difference in adherence or tolerability of different iron-whey-protein formulation doses. Ferritin, haemoglobin and energy levels increased significantly over 12 weeks.

**Conclusion:**

Undiagnosed iron deficiency is common in women of childbearing age with a history of intolerance to oral iron and iron-whey-protein microspheres can improve adherence, GI tolerability, iron stores, haemoglobin and energy levels in these women.

**Clinical trial registration:**

Clinicaltrials.gov identifier (registration includes full trial protocol): NCT04778072.

**Supplementary Information:**

The online version contains supplementary material available at 10.1007/s11096-023-01640-7.

## Impact statements

- Pharmacists are highly accessible healthcare professionals to women of childbearing age and have an important role to play in their care.
- Screening for oral iron intolerance amongst these women can identify a high risk cohort for iron deficiency.
- These women were adherent to Iron-whey-protein microspheres up to 50 mg daily, also demonstrating improved iron stores and haemoglobin.

## Introduction

Women of childbearing age are at high-risk of low iron stores, iron deficiency and anaemia due to inadequate iron intake and/or menstrual blood loss [1, 2]. These conditions are frequently managed using oral iron supplementation. However, due to the low fractional absorption of oral iron [3], very high doses (e.g. ferrous sulfate at 100–200 mg elemental iron daily) are often recommended by healthcare professionals including pharmacists [4], yet systematic evaluation shows adverse gastrointestinal (GI) effects in up to 90% of patients, particularly with ferrous sulfate and ferrous fumarate [5–8]. This results in poor adherence, which may perpetuate iron deficiency and the development of anaemia in vulnerable women [6, 9]. Slow progress on the World Health Organization (WHO) goal to achieve a 50% reduction of anaemia among women of reproductive age (15–49 years) by 2025 [10] has been linked to poor oral iron adherence in a recent International Pharmaceutical Federation report, which also highlights the important role of pharmacists as accessible professionals supporting these women [11].

Despite the recognition of adverse GI effects of oral iron supplementation, there is a lack of research on the prevalence of low iron stores (ferritin < 30 µg/L) in consecutive, consenting, adult women of childbearing age with self-reported adverse GI effects while taking oral iron. Some researchers have advocated for intravenous iron as an efficacious solution to the needs of women with intolerance to oral iron [12]. However, iron infusions require resource-intensive administration as well as monitoring in an outpatient healthcare setting, and 1 in 10 patients may also experience infusion reactions [12, 13]. Adverse GI effects are attributed to iron damage to the intestinal mucosa, in part due to oxidative stress, demonstrated with ferrous sulfate [14–16], and are dose-related [3, 6, 17]. Enteric coated or delayed release formulations of ferrous sulfate can, in principle, address upper GI adverse effects (e.g. nausea, abdominal pain, heartburn, eructation), yet they can also reduce absorption and potentially aggravate lower GI effects (e.g. constipation, diarrhoea), further compromising adherence and absorption [18, 19]. A systematic review of oral iron treatment studies in women of childbearing age, predominantly looking at ferrous sulfate, concluded that there is heterogeneity, bias and imprecision in the reporting of adverse GI symptoms with iron treatment [8]. The Gastrointestinal Symptom Rating Scale (GSRS), which was developed and validated as a reliable screen for GI disorders, could help to standardise evaluation of GI symptoms amongst oral iron users [20–22].

A formulation of ferrous iron in a de-calcified, denatured whey protein matrix formulation at a daily elemental iron dose of 25 mg, has previously reported improved bioavailability and reduced iron induced oxidative stress in gut intestinal epithelial cell lines in-vitro [23]. This suggests that the formulation may have value in managing iron deficiency in women with oral iron intolerance, although to date there are no prospective, comparative clinical data on different doses over time.

### Aim

The present study aimed to understand the prevalence of iron deficiency in women of childbearing age reporting intolerance to oral iron and the tolerability of three doses of an iron-whey-protein formulation in the care of these women.

### Ethics approval

The study was approved by the Cork University Hospital Research Ethics Committee (AFCRO 080) on 10/7/2018, conformed to the principles of the Declaration of Helsinki and all women provided written informed consent.

## Method

### Screening procedure for study participants

Non-pregnant women of childbearing age (18–55 years) with a self-reported history of intolerance to oral iron, without a current diagnosis of gastrointestinal disease, underwent an initial phone screen to confirm their demographics, medical history and history of gastrointestinal intolerance to oral iron. This included the administration of the GSRS questionnaire over the phone in order to assess any GI symptoms. These women were contacted following responses to adverts in local newspapers, an online parenting website (www.rollercoaster.ie), general practitioners' offices, pharmacies and the Atlantia Clinical Research Organisation database. Following a washout (≥ 1 week) of current oral iron, 203 women were screened in the clinic for iron deficiency, anaemia and repeat of the GSRS questionnaire in order to assess GI symptoms. Women with iron deficiency (ferritin < 30 µg/L) were invited to participate in the prospective, randomised, controlled trial. Consecutive, consenting, women were included in stratified groups with or without anaemia (haemoglobin < 12.5 g/dL).

Excluded were women taking concurrent medication which interfered with the absorption of iron (e.g. tetracyclines, calcium supplements), a history of dairy allergy or hypersensitivity to any of the components of the test product, those with severe anaemia (haemoglobin < 9.5 g/dL) and women suffering from any condition which contraindicated, in the investigator’s judgement, entry to the study.

### Randomised, controlled, trial and outcome measures

Included women were randomised in a prospective, double-blind, parallel group, clinical study of Iron-Whey Protein microspheres (IWP, Active Iron®) at three different elemental iron doses: a high dose group (25 mg capsule twice daily); a standard dose group (25 mg in the morning with matching dummy capsule in the evening), a low dose group (14 mg in the morning and matching dummy capsule in the evening). Women were instructed to take IWP orally on an empty stomach and this was confirmed at clinic and phone visits. Details of the previous oral iron product were documented at baseline visit. Changes in adherence were measured using medication-possession-ratio and adverse GI effects using self-report and GSRS at 6 and 12 weeks were documented. Changes in iron stores, haemoglobin and energy levels were also analysed. A minimum of 11 women per dose group were required based on a pilot survey and assumption that the adherence rate of a population of women with a self-reported history of intolerance to oral iron will increase from 50 to 80%, with a two-sided type I error rate of 5% (*α* = 0.05) and power, 1−*β* = 0.90. In addition, 8 women with anaemia are required to detect an increase of 1.0 g/dL in haemoglobin, with standard deviation (*σ*) = 1.0 g/dL, a two-sided type I error rate of 5% (*α* = 0.05) and power, 1−*β* = 0.80. More details of screening procedures, sample size calculations, treatment blinding, randomisation procedures and statistical analyses are presented in the Supplemental File.

Descriptive statistics used one way ANOVA or Kruskall-Wallis for comparison across three dose groups, *T*-tests for pairwise comparisons of normally distributed variables and Wilcoxon tests for pairwise comparisons of non-normally distributed variables. The primary endpoint of the prospective randomised controlled study was the difference in proportion of subjects persistent and adherent (≥ 80% medication possession ratio, based on pill counts) compared to the prior iron product used, measured in pre and post analyses using Fisher exact test. Women were classified as non-adherent if they had medication possession ratio < 80% or were non-persistent. Women lost to follow up were assumed to be non-adherent. Medication possession ratio was evaluated at week 6 and week 12 and averaged to produce an overall score. Secondary endpoints were the change in haemoglobin, ferritin and transferrin saturation levels and the self-reported upper and lower GI tolerability and GSRS over the 12 week period. Haemoglobin was assessed in the overall cohort and in the pre-specified stratified subgroup with anaemia (Hb < 12 g/dL). In an exploratory analysis, health related quality of life was analysed using the Short Form (SF)-36 questionnaire (Rand Corporation https://www.rand.org/health-care/surveys_tools/mos/36-item-short-form.html, accessed 12th October 2022). Primary and secondary outcome measures were also analysed with adjustment for the effects of baseline age, body mass index and systolic blood pressure using linear and logistic regression. All analyses were two-tailed and a *P*-value of < 0.05 was considered statistically significant. All analyses were carried out using *R* version 4.0.1 (2020).

## Results

### Screening enrolment and randomisation

**Fig. 1:**
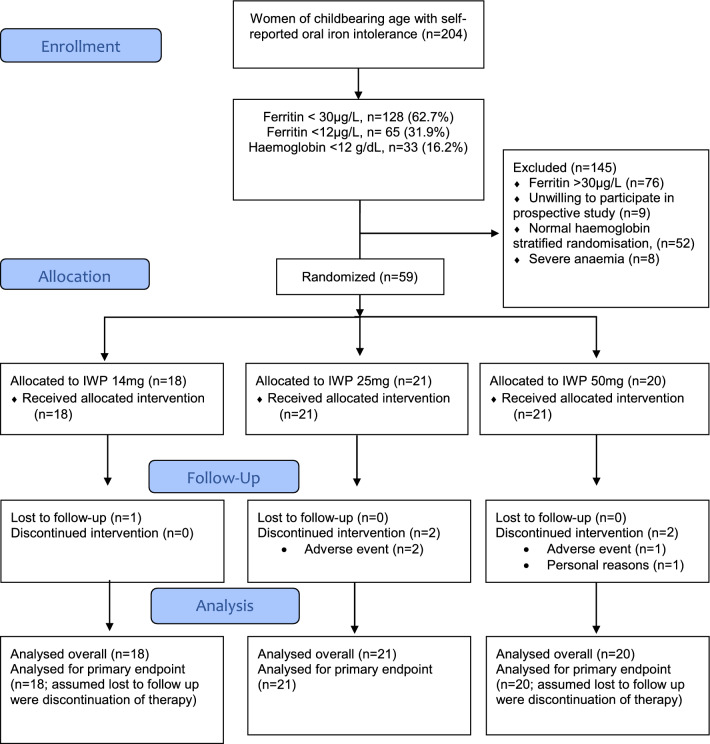
CONSORT Flow Diagram for the PRECISION study. A total of 52 women were not included in the prospective treatment study because they had iron deficiency without anaemia and the stratified quota of 30 women had already been reached. A further 8 were excluded because of severe anaemia and 9 did not want to participate in the prospective study for personal reasons. Participants in the prospective study were also more likely to have a history of iron deficiency and anaemia than the original screened cohort.

The CONSORT diagram for the overall study is presented in Fig. 1. Between the 8th October 2018 and the 17th August 2020, 204 consecutive adult women of childbearing age with a history of gastrointestinal intolerance to oral iron were screened in Cork, Ireland. Of these, almost two in three had low iron stores or iron deficiency (ferritin < 30 µg/L, *n* = 128, 62.7%). A total of 33 (16.2%) had anaemia (haemoglobin < 12 g/dL). Moderate to severe iron deficiency (ferritin cut-off 12 µg/L) affected 65 (31.9%) women and 24 of these also had anaemia. Details of the screened population according to ferritin levels ≥ or < 30 µg/L are presented in Table 1. Complete blood counts were available in 123 (60.3%) of the screened population and this subgroup showed similar levels of low iron stores (*n* = 82, 66.7%), associated with abnormal red blood cell indices (haemoglobin, haematocrit, mean corpuscular haemoglobin, mean corpuscular haemoglobin concentration and higher red blood cell distribution width). Only 17 (8.3%) of those with ferritin < 30 µg/L self-reported a history of iron deficiency and 26 (16.9%) reported a history of iron deficiency and/or anaemia.

**Table 1:** Demographic, anthropomorphic, iron, haemoglobin and full blood count profile of adult women of childbearing age with a self-reported gastrointestinal intolerance to oral iron screened for our study

	All participants *N* = 204	Normal ferritin ($\ge 30$ µg/L), *N* = 76	Low ferritin (< 30 µg/L), *N* = 128	*P* value*
Age, years	36.6 ± 10.1	36.8 ± 10.0	36.6 ± 10.2	0.911
History of iron deficiency, *n* (%)	17 (8.33)	4 (5.26)	13 (10.2)	0.337
History of anaemia, *n* (%)	23 (11.3)	6 (7.89)	17 (13.3)	0.249
History of iron deficiency or anaemia, *n* (%)	26 (12.7)	8 (10.5)	18 (14.1)	0.606
Weight, kg	70.7 (60.7; 81.4)	70.4 (60.9; 83.1)	71.3 (60.6; 79.7)	0.784
Height, m	1.65 ± 0.06	1.65 ± 0.06	1.65 ± 0.06	0.756
BMI, kg/m^2^	25.8 (22.3; 29.6)	25.5 (22.3; 30.4)	25.9 (22.2; 29.3)	0.962
SBP, mmHg	109 (102; 117)	109 (102; 117)	109 (102; 117)	0.900
DBP, mmHg	73.8 ± 9.38	73.8 ± 8.24	73.9 ± 10.0	0.923
HR, bpm	70.0 (65.0; 77.0)	69.5 (64.0; 76.0)	70.0 (65.0; 77.0)	0.640
*Smoking status*				0.890
Never smoked, *n* (%)	133 (65.2)	49 (64.5)	84 (65.6)	
Previous smoker, *n* (%)	47 (23.0)	17 (22.4)	30 (23.4)	
Current smoker, *n* (%)	24 (11.8)	10 (13.2)	14 (10.9)	
Alcohol consumption, units/week	2.6 ± 3.0)	2.9 (3.3)	2.5 (2.8)	0.345
Depot contraceptive, *n* (%)	8 (3.9)	4 (5.3)	4 (3.12)	0.474
Patch or ring contraceptive, *n* (%)	15 (7.4)	9 (11.8)	6 (4.7)	0.106
Oral contraceptive, *n* (%)	37 (18.1)	15 (19.7)	22 (17.2)	0.788
Serum Iron, µmol/L	15.8 ± 8.35	20.9 ± 7.38	13.4 ± 7.72	< 0.001
Total iron binding concentration, µmol/L	61.0 ± 9.94	54.4 ± 8.73	64.0 ± 8.96	< 0.001
Transferrin saturation, %	27.1 ± 15.2	38.8 ± 13.3	21.7 ± 12.9	< 0.001
Ferritin, µg/L	18.0 (9.00; 43.2)	50.5 (41.0; 66.0)	11.5 (7.00; 15.8)	< 0.001
Hb, g/dL	12.9 ± 1.16	13.4 ± 0.75	12.7 ± 1.28	< 0.001
Haematocrit, L/L	0.40 (0.37; 0.42)	0.41 (0.40; 0.42)	0.38 (0.36; 0.41)	< 0.001
Mean cell volume, fL	88.8 (85.4; 92.1)	91.9 (87.8; 94.8)	88.3 (84.3; 90.4)	< 0.001
Mean cell Hb, pg	28.9 (27.3; 30.4)	29.8 (28.5; 31.3)	28.5 (26.6; 29.9)	< 0.001
Mean cell Hb concentration, g/dL	32.4 ± 1.19	32.8 ± 1.20	32.2 ± 1.14	0.009
Red cell distribution width, %	13.3 (12.8; 14.2)	12.9 (12.6; 13.4)	13.6 (13.0; 14.7)	< 0.001
White cell count, 10^−9^/L	5.60 (4.76; 6.79)	5.73 (5.17; 7.05)	5.58 (4.55; 6.39)	0.111
Red cell count, 10^−12^/L	4.42 (4.24; 4.70)	4.43 (4.27; 4.80)	4.41 (4.18; 4.66)	0.418
Platelets, 10^−9^/L	287 (247; 324)	298 (270; 324)	278 (243; 323)	0.196
Neutrophils, 10^−9^/L	3.26 (2.49; 4.21)	3.48 (2.83; 4.47)	3.20 (2.44; 4.11)	0.202
Lymphocytes, 10^−9^/L	1.73 (1.36; 2.06)	1.84 (1.43; 2.11)	1.67 (1.33; 1.88)	0.046
Monocytes, 10^−9^/L	0.44 (0.35; 0.53)	0.45 (0.34; 0.56)	0.44 (0.36; 0.52)	0.718
Eosinophils, 10^−9^/L	0.14 (0.08; 0.26)	0.16 (0.10; 0.27)	0.12 (0.06; 0.25)	0.076
Basophils, 10^−9^/L	0.03 (0.02; 0.04)	0.03 (0.02; 0.05)	0.02 (0.02; 0.04)	0.218

Women are presented with ferritin levels $\ge$ or < 30 µg/L. Unless otherwise stated, data are presented as mean ± standard deviation or median (interquartile range)

*T-tests for normally distributed variables, Wilcoxon test for non-normal distributions and Fisher’s Exact tests for categorical comparisons

*SBP* Systolic blood pressure, *DBP* Diastolic blood pressure, *HR* Heart rate, *bpm* Beats per minute, *BMI* Body mass index, *Hb* Haemoglobin

Recruitment to the prospective treatment study was stratified by anaemia status (maximum 30 participants with Hb < or ≥ 12 g/dL). Excluded (*n* = 145) were 76 women with normal ferritin levels (> 30 µg/L), 8 with severe anaemia and 52 with iron deficiency and normal haemoglobin (due to stratified randomisation). In addition, a further 9 women were not willing to participate in the prospective study for personal reasons. Randomisation was carried out by a statistician employed by Atlantia CRO and none of the study team or investigators had access to the randomisation code. Further details of the investigational product and blinding is provided in the Supplemental File. Three people were lost to follow up and 2 discontinued the intervention. However, the primary endpoint was analysed for all 59 participants on the assumption that those lost to follow up and those who discontinued were non-adherent (assumed medication possession ratio = 0). Due to the onset of COVID-19 pandemic and subsequent lockdowns, only 40 participants were able to attend the clinic for follow-up blood assessments. The study completed when the last participant was followed up. Last participant last visit was 10th November 2020.

### Baseline characteristics of the prospectively studied population

The baseline characteristics of the randomised, controlled, trial population and profile of adverse GI effects of prior iron products are presented in Table 2. The prior iron products and dose groupings are detailed in Supplemental File Table S1. Most participants (7 in 10) were taking high dose ferrous iron products and the most common oral iron adverse GI effect reported at baseline was constipation (affecting 3 in 4 women), followed by abdominal pain (affecting 1 in 2 women) and nausea (affecting 1 in 3 women). Following an average washout period of 9.8 ± 3.9 days, the overall GSRS score was significantly reduced in the cohort at the prospective study baseline (19.4 ± 7.05) compared with the previous iron product (30.6 ± 9.71, *T*-test *P* < 0.001 vs baseline).

**Table 2:** Demographic, anthropomorphic, iron, haemoglobin and full blood count profile of participants with ferritin < 30 µg/L and self-reported gastrointestinal intolerance to oral iron who were randomised to three different daily elemental iron doses of IWP (14 mg, 25 mg, 50 mg)

	All Women *N* = 59	IWP 14 mg *N* = 18	IWP 25 mg *N* = 21	IWP 50 mg *N* = 20
Age, years	35.2 ± 11.0	35.3 ± 11.8	34.0 ± 10.0	36.1 ± 11.7
SBP, mmHg	109 (104; 119)	116 (108; 123)	105 (103; 114)	110 (100; 122)
DBP, mmHg	74.7 ± 9.49	75.3 ± 8.90	75.1 ± 8.34	73.7 ± 11.4
HR, bpm	70.3 ± 9.88	70.2 ± 10.4	70.6 ± 10.3	70.0 ± 9.47
Weight, kg	72.4 (59.6; 82.4)	75.5 (60.8; 85.6)	72.4 (62.4; 78.2)	69.7 (57.0; 79.2)
BMI, kg/m^2^	26.4 (22.0; 30.6)	27.1 (22.2; 31.6)	27.6 (22.6; 30.2)	25.1 (20.5; 27.3)
Alcohol consumption, units/week	3.55 ± 3.09	3.07 ± 2.94	4.40 ± 3.62	3.13 ± 2.72
*Smoking status*				
Never smoked, *n*(%)	9 (15.3)	1 (5.6)	5 (23.8)	3 (15.0)
Previous smoker, *n*(%)	40 (67.8)	15 (83.3)	13 (61.9)	12 (60.0)
Current smoker, *n*(%)	10 (16.9)	2 (11.1)	3 (14.3)	5 (25.0)
Days since screening visit	9.83 ± 3.9	10.1 ± 4.0	9.29 ± 2.9	10.2 ± 4.7
Serum Iron, µmol/L	9.60 (5.75; 16.3)	12.6 (5.73; 18.5)	10.3 (7.60; 14.8)	8.30 (5.45; 14.5)
Unbound iron binding concentration, µmol/L	52.2 ± 13.3	52.2 ± 14.5	51.5 ± 11.0	53.1 ± 15.0
Total iron binding concentration, µmol/L	65.0 (56.6; 69.8)	67.3 (56.6; 70.7)	62.9 (56.8; 68.8)	65.2 (56.0; 69.2)
Transferrin saturation, %	16.1 (9.00; 27.4)	19.4 (7.60; 30.2)	16.6 (11.0; 22.8)	13.2 (8.70; 22.0)
Ferritin, µg/L	9.00 (6.00; 15.5)	7.50 (6.00; 10.8)	8.00 (7.00; 12.2)	13.0 (6.00; 20.8)
White cell count, 10^−9^/L	5.26 (4.34; 6.67)	5.73 (4.50; 6.66)	5.27 (4.37; 6.74)	4.92 (3.96; 6.53)
Red cell count, 10^−12^/L	4.41 ± 0.36	4.41 ± 0.34	4.41 ± 0.37	4.42 ± 0.40
Hb, g/dL	12.3 ± 1.25	12.0 ± 1.44	12.3 ± 1.27	12.4 ± 1.07
Haematocrit, L/L	0.38 ± 0.03	0.38 ± 0.03	0.38 ± 0.03	0.38 ± 0.03
Mean cell volume, fL	87.6 (83.8; 89.9)	86.8 (79.9; 89.9)	87.0 (84.9; 89.6)	88.7 (86.3; 91.8)
Mean cell Hb, pg	28.2 (26.4; 29.5)	27.9 (25.5; 29.3)	27.8 (27.3; 29.1)	28.6 (27.8; 30.3)
Mean cell Hb concentration, g/dL	32.1 ± 1.32	32.0 ± 1.51	32.1 ± 1.15	32.3 ± 1.34
Red cell distribution width, %	13.8 (13.1; 14.6)	13.8 (13.1; 15.4)	14.1 (13.0; 14.5)	13.7 (13.1; 14.6)
Platelets, 10^−9^/L	288 (229; 334)	296 (260; 328)	289 (204; 333)	264 (230; 339)
Neutrophils, 10^−9^/L	2.87 (2.33; 3.98)	3.09 (2.39; 3.73)	2.94 (2.46; 4.52)	2.64 (2.09; 3.86)
Lymphocytes, 10^−9^/L	1.68 ± 0.48	1.91 ± 0.52	1.63 ± 0.39	1.51 ± 0.48
Monocytes, 10^−9^/L	0.42 (0.33; 0.55)	0.40 (0.31; 0.49)	0.42 (0.36; 0.55)	0.42 (0.33; 0.56)
Eosinophils, 10^−9^/L	0.11 (0.08; 0.21)	0.11 (0.06; 0.31)	0.10 (0.08; 0.16)	0.14 (0.09; 0.23)
Basophils, 10^−9^/L	0.02 (0.02; 0.04)	0.02 (0.02; 0.04)	0.02 (0.02; 0.03)	0.04 (0.02; 0.05)
*Self-reported adverse GI effects with previous oral iron product*				
Constipation, *n*(%)	44 (74.6)	11 (61.1)	18 (85.7)	15 (75.0)
Diarrhoea, *n*(%)	4 (6.8)	1 (5.6)	2 (9.5)	1 (5.0)
Abdominal pain, *n*(%)	29 (49.2)	10 (55.6)	10 (47.6)	9 (45.0)
Nausea, *n*(%)	22 (37.3)	6 (33.3)	8 (38.1)	8 (40.0)
Vomiting, *n*(%)	1 (1.7)	0 (0.0)	0 (0.0)	1 (5.0)
Indigestion, *n*(%)	12 (20.3)	5 (27.8)	4 (19.0)	3 (15.0)
Heartburn, *n*(%)	7 (11.9)	3 (16.7)	3 (14.3)	1 (5.0)
Total number of adverse GI events reported on prior product	4.0 ± 2.2	3.8 ± 2.4	4.7 ± 2.5	3.4 ± 1.6
Persistent with prior product, *n* (%)	12 ± 20.3	4 ± 22.2	5 ± 23.8	3 ± 15.0
GSRS score on prior product	30.6 ± 9.7	29.9 ± 8.4	31.3 ± 8.4	30.4 ± 12.3
GSRS score following washout	19.4 ± 7.01	20.7 ± 7.4	19.4 ± 67	18.4 ± 7.3

Unless otherwise stated, data are presented as mean ± standard deviation or median (interquartile range). The self-reported gastrointestinal adverse effect profile and Gastrointestinal Symptom Rating Score (GSRS) associated with previous oral iron products are presented. An overall GSRS gut symptom score of 15 is a perfect GSRS score reflecting no adverse GI symptoms. The majority of the cohort (*n* = 40, 69.5%) had previously been taking high dose of oral iron (> 60 mg elemental Iron)

*IWP* Iron-whey-protein formulation, *SBP* Systolic blood pressure, *DBP* Diastolic blood pressure, *HR* Heart rate, *bpm* Beats per minute, *BMI* Body mass index, *Hb* Haemoglobin. *GI* Gastrointestinal, *GSRS* Gastrointestinal Symptom Rating Score

### Adherence/persistence

**Fig. 2:**
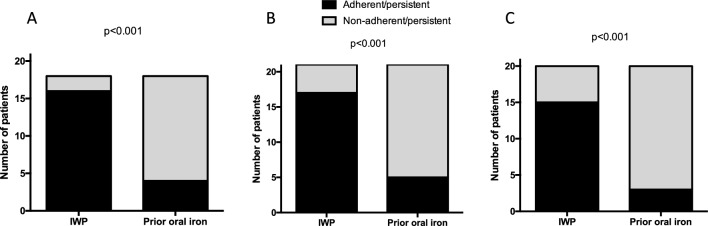
Overall adherence/persistence with IWP amongst 59 women with a history of intolerance to oral iron and with low iron, moderate to severe iron deficiency or iron deficiency anaemia. The odds ratio (OR) for improvement in adherence/persistence was consistent across the three dose groups: 4.0 (95% CI 1.7 to 9.6) for IWP 14 mg (Fig. 2A); 3.4 (95% CI 1.5 to 7.5) for IWP 25 mg (Fig. 2B); 5.0 (95% CI 1.7 to 14.6) for IWP 50 mg (Fig. 2C), Logistic regression, all *P* < 0.05)

In the 12-week interventional study, 3 participants stopped treatment due to possible (not probable) adverse effects, 1 participant withdrew for personal reasons (related to menorrhagia) and one participant was lost to follow-up following the baseline visit. For the purpose of the primary analysis, it was assumed that these 5 participants were non-adherent and non-persistent. A total of 48 (81.4%) participants were classified as adherent/persistent with therapy using IWP compared to 12 (20.3%) taking the prior oral iron (Fisher’s Exact test, *P* < 0.001). A total of 16 (88.9%), 17 (80.1%) and 15 (75.0%) participants taking 14 mg, 25 mg and 50 mg respectively were adherent/persistent with the therapy. These were significantly higher than the 4 (22.2%), 5 (23.8%) and 3 (15.0%) participants who persisted taking the previous oral iron in the respective 14 mg, 25 mg and 50 mg groups (Fisher’s Exact test, all *P* < 0.001 versus IWP) (Fig. 2). Overall, patients taking IWP were more likely (OR 4.0 (95% CI 2.4 to 6.7) to be adherent/persistent with IWP than with the previous oral iron (Fisher’s Exact Test, *P* < 0.001).

Median adherence with IWP was 96.4% (IQR 83.6, 100.6) over the course of 12 weeks and did not differ across the three dose groups (Supplemental File Figure S1). This includes a medication possession ratio expressed as % using pill-counts of 0 attributed to 5 women who withdrew from the study following randomisation. Of the remaining 54 women who persisted with therapy, 48 demonstrated good average adherence (≥ 80% medication possession ratio, based on pill counts). There was no association between IWP dose and average medication-possession-ratio (OR 0.99 (95%CI 0.61 1.58), *P* = 0.67) or overall adherence > 85% (OR 1.00 (95%CI 0.99 1.01), *P* = 0.81) when adjusted for baseline age, SBP, BMI using linear and logistic regression.

### Elicited adverse gastrointestinal effects and gastrointestinal symptom rating scale

**Fig. 3:**
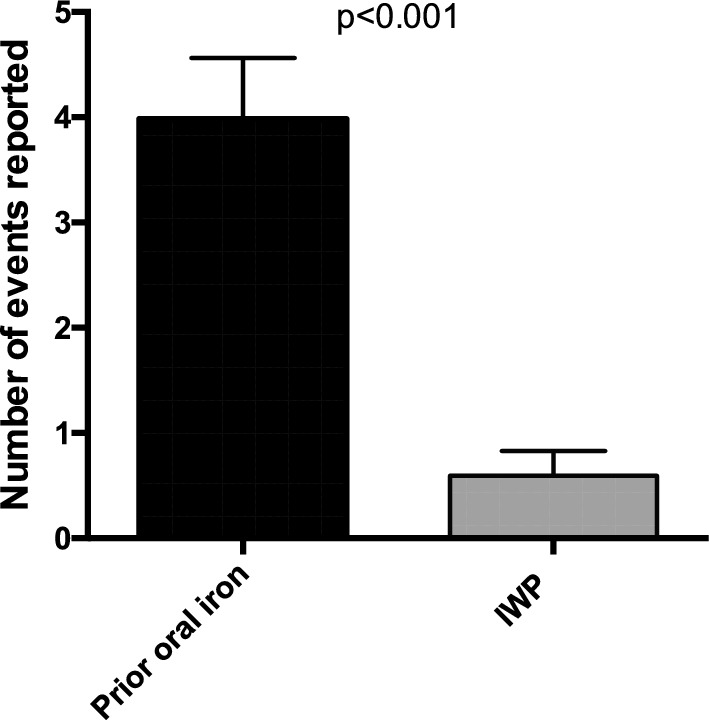
Number of elicited adverse GI events reported with previous oral iron product in comparison with IWP amongst 59 women with a history of intolerance to oral iron with low iron, moderate to severe iron deficiency or iron deficiency anaemia. Overall, using logistic regression, women were 4.0 (95%CI 2.3 to 7.0, *P* < 0.001) more likely to experience constipation and 3.2 (95%CI 1.7 to 6.2, *P* < 0.001) more likely to experience abdominal pain with the prior oral iron product than with IWP. *IWP* Iron-whey-protein formulation

Participants in the prospective study attributed an average of 4.0 ± 2.2 adverse GI effects to the prior oral iron product. Participants reported six times fewer adverse events that were possibly or probably associated with IWP (0.59 ± 0.91, *T*-test *P* < 0.001 versus prior oral iron product, Fig. 3). In accordance with these data, the overall GSRS score did not change from baseline (19.4 ± 7.1) to 6 weeks (21.6 ± 8.7) and 12 weeks (21.2 ± 7.5) post randomisation for the entire cohort (ANOVA, *P* = 0.33, Supplemental File Figure S2). Average GSRS on treatment with IWP was the same across the three dose groups: 20.2 ± 6.6 in the 14 mg daily dose group, 22.0 ± 6.6 in the 25 mg daily dose group and 21.1 ± 9.1 in the 50 mg daily dose group (ANOVA, *P* = 0.78). The average GSRS on treatment was strongly associated with the GSRS reported on prior iron products (OR 1.35 (95%CI 1.13 1.61)), but not with IWP dose group (OR 0.99 (95%CI 0.88 1.11)) using linear regression with adjustment for baseline age, SBP, BMI. A total of 44 (74.6%) and 29 (49.2%) women had reported constipation and abdominal pain respectively attributed to the previous oral iron product. This was reduced to 11 (18.6%) and 9 (15.3%) respectively with IWP. Four women reported diarrhoea with the previous oral iron product compared with 2 women taking IWP.

### Effects on ferritin, transferrin saturation, haemoglobin and energy/fatigue levels

**Fig. 4:**
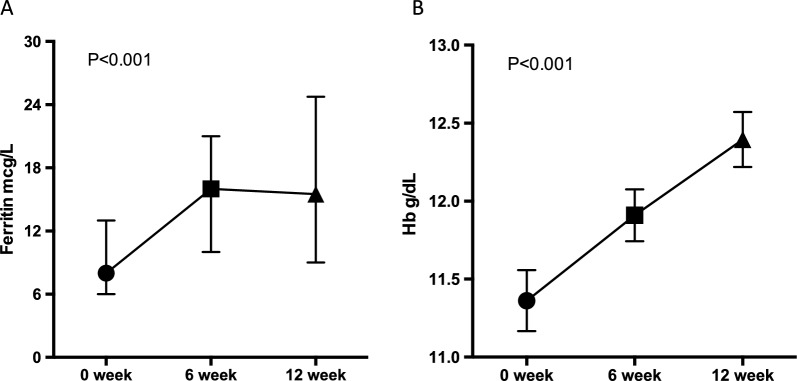
Median ferritin **A** levels in women with iron deficiency (*n* = 40) and mean haemoglobin **B** levels over time in women with iron deficiency anaemia (*n* = 21). The mean ferritin increases within dose groups over 12 weeks using T-test were: 14 mg daily dose group 1.6 (95% CI − 1.4 to 4.6, *P* = 0.33) µg/L; the 25 mg daily dose group 6.6 (95% CI 2.5 to 10.7, *P* = 0.004) µg/L and 50 mg daily dose group 9.3 (95% CI 3.8 to 14.8, *P* = 0.002) µg/L. The mean increases in haemoglobin over 12 weeks within dose groups using T-tests were: IWP 14 mg daily dose group, 0.66 g/dL (95% CI -0.84 to 2.16, *P* = 0.43); IWP 25 mg daily dose group (0.86 g/dL (95% CI 0.38 to 1.33, *P* = 0.004); IWP 50 mg daily dose group, 1.35 g/dL (95% CI 0.54 to 2.16, *P* = 0.006). *IWP* Iron-whey-protein formulation, *Hb* Haemoglobin

Median ferritin levels overall increased from 8.00 (IQR 6.00; 13.0) to 15.5 (IQR 9.00;24.2) µg/L at 12 weeks over the study (Kruskal–Wallis test, *P* < 0.001, Fig. 4A). More detailed analysis of iron parameters in the IWP dose group is presented in Table 3, where within-group changes in ferritin and haemoglobin levels at 12 weeks were significant in the 25 mg and 50 mg dose groups only.

**Table 3:** Detailed serum iron, transferrin saturation and ferritin in the overall cohort and in the three dose groups

	All participants *N* = 40	IWP 14 mg *N* = 12	IWP 25 mg *N* = 14	IWP 50 mg *N* = 14	*P*-value
Serum Iron baseline, µmol/L	11.3 (7.45)	12.5 (7.42)	11.2 (6.63)	10.3 (8.58)	0.774
Serum Iron 6w, µmol/L	17.4 (9.10)*	18.3 (12.9)*	17.8 (7.91)*	16.1 (6.56)*	0.814
Serum Iron 12w, µmol/L	20.5 (11.0)**	22.4 (13.1)*	20.6 (11.0)*	18.7 (9.52)**	0.700
TSAT baseline, %	18.4 (13.4)	20.6 (12.9)	17.7 (11.8)	17.2 (15.8)	0.795
TSAT 6w, %	28.5 (13.8)**	28.8 (17.4)**	30.5 (14.1)**	26.3 (10.2)**	0.726
TSAT 12w, %	33.6 (17.6)***	35.1 (19.3)**	34.8 (19.5)**	31.2 (14.9)***	0.829
Ferritin baseline, µgl/L	8.00 (6.00; 13.0)	8.00 (5.00; 10.2)	8.00 (7.00; 12.2)	9.00 (5.25; 17.5)	0.862
Ferritin 6w, µgl/L	17.0 (10.8; 22.0)*	10.5 (7.00; 15.8)	17.5 (13.8; 21.8)**	20.0 (12.5; 24.2)**	0.037
Ferritin 12w, µgl/L	15.5 (9.00; 24.2)*	8.50 (6.50; 16.2)	16.0 (11.2; 22.5)**	20.0 (12.2; 30.0)**	0.013
Hb baseline, µgl/L	11.3 (0.98)	11.0 (1.26)	11.3 (0.81)	11.4 (0.76)	0.223
Hb 6w, µgl/L	11.9 (0.76)*	11.4 (0.72)	11.7 (0.70)	12.5 (0.47)*	0.012
Hb 12w, µgl/L	12.4 (0.81)**	11.9 (0.66)	12.3 (0.97)*	12.8 (0.53)**	0.075

Haemoglobin data are presented in the subgroup with iron deficiency anaemia

All data quoted as mean ± standard deviation for normal distributions or median (interquartile ranges) for non-normal distributions. Comparisons across dose groups are made using ANOVA and Kruskal–Wallis for normal and non-normal distributions respectively (*P*-values quoted in the Table). Within group comparisons versus baseline use *T*-tests and Wilcoxon tests for normal and non-normal distributions respectively and are denoted as follows; *P < 0.05; ** *P* < 0.01

*IWP* Iron-whey-protein formulation, *TSAT* Transferrin saturation, 6w 6-week, 12w 12 week

The within-group increases in ferritin (all T-tests) were 1.6 µg/L (95% CI − 1.4 to 1.4, *P* = 0.26) in the 14 mg dose group, 6.6 µg/L (IQR 2.5 to 10.7, *P* = 0.0042) in the 25 mg dose group and 9.3 µg/L (IQR 3.8 to 14.8, *P* = 0.0028) in the 50 mg dose group. Multivariable linear regression showed that changes in ferritin levels over 12 weeks were independently associated with IWP doses (OR 1.29 (95%CI 1.11, 1.51) and baseline ferritin level (OR 0.67 (95%CI 0.51, 0.89) with adjustment for baseline age, SBP and BMI. Transferrin saturation values increased during the study period within all dose groups, but were not significantly different across the three dose groups (Table 3). In participants with anaemia, haemoglobin levels increased from 11.36 g/dL (95% CI 10.95 to 11.77) to 12.40 g/dL (95% CI 12.03 to 12.76, *T*-test, *P* < 0.001, Fig. 4B). The within-group increases (all *T*-tests) were 0.56 g/dL (95% CI − 0.61 to 1.73, *P* = 0.26) in the 14 mg dose group, 0.84 g/dL (95% CI 0.27 to 1.42, *P* = 0.016) in the 25 mg dose group and 1.35 g/dL (95% CI 0.54 to 2.16, *T*-test, *P* < 0.01) in the 50 mg dose group (Table 3). Multivariable linear regression showed that change in haemoglobin levels over 12 weeks was not independently associated with IWP doses (OR 1.02 (95% CI 1.00, 1.03, *P* = 0.08) with adjustment for baseline age, SBP, BMI and baseline haemoglobin level (Supplemental File Table S2).

The SF-36 Energy/Fatigue domain scores in the population at baseline (60.9 ± 3.4%) were significantly impaired compared to all the other domain scores at baseline (*T*-test, all *P* < 0.001, Supplemental File Figure S3) and were similar in participants with low iron stores (ferritin 12–30 µg/L, SF-36 Energy/Fatigue 61.1 ± 5.1%) and those with moderate to severe iron deficiency (ferritin < 12 µg/L, SF-36 Energy/Fatigue 60.9 ± 4.5%). These scores increased significantly in the overall group over the study period reaching scores of 71.2 ± 2.6% (*T*-test, *P* < 0.001) and significant within group changes were observed in the 25 mg and 50 mg daily dose groups (Supplemental File Figure S4).

## Discussion

Amongst women of childbearing age, screened principally based on a history of GI intolerance to iron, a majority (62%) had low iron stores, 3 in 10 had moderate to severe iron deficiency, 1 in 6 had iron deficiency anaemia. However, only 1 in 10 of those included in the study reported a formal diagnosis of iron deficiency and/or anaemia. These results are in accordance with findings on iron deficiency and iron deficiency erythropoiesis in adult women who are frequent blood donors [24] as well as in reports focused on the challenge of oral iron adverse effects and adherence [7, 8, 11]. Poor adherence to and lack of persistence with conventional, predominantly high dose, oral iron products was reported in 8 out of 10 women in our study. The study also shows that most of these women were adherent to IWP and there was no difference in adherence or tolerability among the different IWP doses. Nor were any dose related differences identified in gastrointestinal tolerability using the GSRS. Ferritin, haemoglobin and energy levels increased significantly over 12 weeks, especially in the 25 mg and 50 mg daily dose groups, showing that this formulation could provide a treatment option for these women.

The adverse GI effect most commonly reported with the prior iron product in this population was constipation (*n* = 44, 75%), in accordance with a previous systematic review and an outpatient pharmacist intervention for iron deficiency anaemia [4, 8], albeit a majority of women in our study also reported combined upper and lower GI adverse effects. The causes of adverse GI effects with oral iron remain poorly understood, yet endoscopic reports show direct damage from iron deposition in the upper GI tract, which may have a contribution from iron redox activity and associated reactive oxygen species generation [14–16, 25]. Pathophysiological shifts in microbiota composition may also contribute to lower intestinal adverse effects, especially at higher elemental iron dose [16, 26]. Intestinal inflammation can impact on oral iron absorption, which is hepcidin regulated [3, 6]. The high prevalence of constipation underlines the difficulty in advocating delayed release or enteric coated oral iron products as a solution to poor GI tolerability, as they have relatively poor absorption [19], increasing the unabsorbed iron load reaching the bowel and therefore potentially aggravating constipation. Treatment of iron deficiency in an iron intolerant group is for these reasons highly challenging.

Iron deficiency anaemia has been conventionally treated with daily oral doses above 65 mg elemental iron daily [7–9, 27]. Although ferrous sulfate has been considered the gold standard oral iron [27], and is poorly tolerated [3, 5, 6], the majority of women in our prospective study had been taking high-dose, immediate release ferrous fumarate, which also has poor GI tolerability based on our study and previous reports [7]. Considering its high bioavailability, IWP was investigated in the dose range 14–50 mg elemental iron daily in the randomised prospective study. This is also, to our knowledge, the first trial to use the validated GSRS gut-symptom-score [22] to prospectively track GI tolerability over time with a specific, oral iron treatment. Women were more likely to continue using IWP than their previous iron product and IWP resulted in a better GSRS, six times fewer elicited adverse GI events and four times better compliance when compared to the womens’ reported experience with prior oral iron. This is in accordance with reduced iron oxidative stress in gut cells with IWP compared to ferrous sulfate previously reported in vitro [23]. There was no difference between the three IWP dose groups (14 mg, 25 mg, 50 mg daily elemental iron) in terms of adherence or tolerability, suggesting that higher IWP doses can be used, particularly in those with iron deficiency anaemia.

This study showed improvements in ferritin, transferrin saturation and haemoglobin levels as well as increase in the SF-36 energy and fatigue domain scores, particularly in the 25 mg and 50 mg dose groups over a 12 week period. Using 50 mg IWP daily in the subset with mild-to-moderate iron deficiency anaemia, haemoglobin significantly increased by 1.35 g/dL over 12 weeks and was normalised in most women. Iron deficiency can affect other organs/tissues, such as hair growth, immune function, skeletal muscles and the heart, long before there is evidence of impaired erythropoiesis [28, 29]. Although some women did respond to IWP at the nutritional reference value (14 mg), the data do not support the use of this dose in women with iron deficiency and it should be reserved for maintenance of normal iron stores.

This prospective study also showed that few women with low iron stores had a prior diagnosis of iron deficiency or anaemia. While many hospital and commercial laboratories use a diagnostic threshold of 12 µg/L, which is highly specific, it is not sensitive enough for the diagnosis of iron deficiency in pre-menopausal women that can be linked to the presence of clinical symptoms [30]. Indeed, the SF-36 data suggest that ferritin levels between 12 and 30 µg/L are associated with significant impairment of energy and increased fatigue. Healthcare professionals, including pharmacists, may be well placed to help address the challenges of low iron in this large population [4, 11].

There are a number of limitations of this study. First, the study was powered to evaluate the tolerability of three different doses of the IWP formulation in women with a history of intolerance to oral iron and changes in haemoglobin of 1.0 g/dL. The study was not powered a priori on other secondary outcomes and the numbers of women completing the blood analysis was limited due to the onset of COVID-19 lockdowns. Second, there is a reliance in the study on self-report of gastrointestinal intolerance and adherence, which risks inaccurate reporting. However, the use of validated GSRS gut symptom scores may have partially mitigated this risk. Also the use of medication-possession-ratio from pill-counts to monitor adherence is imperfect and during COVID-19 lockdowns, we required women to retain the used blister for subsequent clinic visits. Third, the population was selected on the basis of a previous negative experience of oral iron, which may have introduced selection bias.

## Conclusion

Pharmacists and other healthcare professionals should be aware that undiagnosed iron deficiency and anaemia are common in women of childbearing age with a history of intolerance to oral iron. This study shows that IWP up to 50 mg daily is well tolerated in this population and that higher doses can improve iron stores and haemoglobin levels while reducing tiredness.

### Supplementary Information

Below is the link to the electronic supplementary material.

Supplementary file1 (DOCX 231 KB)
