# Supplementary material for: Management of iron deficiency in women of childbearing age with oral iron intolerance: a prospective, randomised, controlled trial of three doses of an iron-whey-protein formulation: Prospective RandomisEd study of women of Childbearing age with gastroInteStinal Intolerance to Oral iroN (PRECISION)
Source: Int J Clin Pharm. 2023 Dec 26;46(2):390–400. doi: 10.1007/s11096-023-01640-7 (PMC10960882; doi:10.1007/s11096-023-01640-7)

**Supplemental File**

**Management of Iron deficiency in Women of Childbearing Age with Oral Iron Intolerance; A Randomised, Prospective, Study of Three Doses of an Iron-Whey-Protein Formulation.**

Prospective RandomisEd study of women of Childbearing age with gastrointeStinal Intolerance to Oral Iron (**PRECISION**).

**Detailed study procedure**

**Ethics approval, consent to participate and consent to publication:** The PRECISION clinical trial was approved by the University College Cork / Cork University Hospital Ethics Committee and conformed to the principles of the Declaration of Helsinki and all women enrolled provided written, informed consent for participation and permission to publish. The corresponding author confirms that the consent of all authors has been provided to publish this manuscript.

**Screening procedures.**

Subjects were recruited through adverts in local newspapers, online parenting website ([www.rollercoaster.ie](http://www.rollercoaster.ie)), general practitioners offices, pharmacies and the Atlantia Clinical Research Organisation database. Subjects underwent an initial phone screen to confirm their demographics, medical history (including absence of an active diagnosis of gastrointestinal disease) and definite history of gastrointestinal intolerance to oral iron. Consenting, eligible subjects were then asked to omit their oral iron product for at least 7 days and were invited to a study screening visit in the clinic to investigate iron stores, haemoglobin, vital signs (blood pressure, heart rate & temperature), anthropometric measurements (weight, height & body mass index), medical history, history of iron deficiency, history of anaemia and self-reported history of intolerance to oral iron products. The self-reported history collected detailed measures of upper GI symptoms (e.g. self-reported history of stomach pain, nausea, vomiting, eructation, heartburn, indigestion) and lower GI symptoms (e.g. bowel habit changes, diarrhoea, constipation, stool discolouration). A fasting blood sample (16mLs) was collected and ferritin and haemoglobin was measured.

Excluded were women taking concurrent medication which interferes with the absorption of iron (e.g. tetracyclines, calcium supplements), those with a history of dairy allergy or hypersensitive to any of the components of the test product, those with severe anaemia (haemoglobin <9.5 g/dL) and a malignant disease or any concomitant end-stage organ disease or significant acute or chronic, unstable and untreated disease or any condition, which contraindicated, in the investigator’s judgement, entry to the study.

**Prospective, randomised, controlled trial procedures**.

The prospective trial evaluated the adherence, tolerability and efficacy of IWP overall and of three different elemental iron doses of IWP in women with a history of intolerance to oral iron, low iron or iron deficiency, with or without anaemia. Randomisation was carried out by a statistician employed by Atlantia CRO using R software and the Randomisation Code was confidentially maintained by the Managing Director of the company until the study was complete and the database locked. None of the study team or investigators had access to the Randomisation Code. Treatment doses were concealed in identical hydroxypropylmethyl cellulose hard capsules along with matching placebos (lactose) for the evening dose for those women taking 14mg and 25mg daily. All capsules were blister-packed in aluminium foil. All women, investigators and personnel involved in the study were blinded to treatment allocation. All treatments were manufactured in a Good Manufacturing Practice facility at R-Pharm, Munich, Germany. Evaluation of women was carried out during three clinic visits (baseline, week 6 and week 12) and two phone call visits (weeks 3 and 9). In addition, there was stratified randomisation by a statistician employed by Atlantia CRO to include up to 30 women with iron deficiency and mild to moderate anaemia as well as up to 30 women with iron deficiency without anaemia. The random allocation sequence was linked to a study number and concealed in the randomisation key. This key was held by the Managing Director of Atlantia CRO before and during the recruitment period and was not available to the study team or investigators at any point before database lock. The Managing Director of Atlantia CRO assigned women to study numbers sequentially, and therefore randomised treatment allocation. The Managing Director of Atlantia CRO had no interaction with women involved in the study and was part of the study team.

Eligible women between 18 and 55 years of age, with a self-reported history of intolerance to oral iron and low iron stores (ferritin < 30 µg/L) or iron deficiency (ferritin < 12 µg/L) with or without mild to moderate anaemia (haemoglobin ≥9.5 g/dL and <12.0 g/dL) were invited to participate in the prospective treatment study. Once the screening blood results were reviewed and eligibility for the prospective study was confirmed following a washout period of at least one week from the previous iron product, the subject returned to the clinic for full clinical assessment. At visit 2 (baseline of the prospective study), a fasting blood sample was collected for evaluation of ferritin, serum iron, unbound iron binding capacity, transferrin saturation, and full blood count (including haemoglobin). A urine sample was collected, and pregnancy test performed. Subjects were queried about any changes in their health status and any non-treatment emergent events since the screening visit and medications were recorded. This included details of the prior oral iron product taken (dose, form, dosage frequency). Self-reported adherence, persistence, upper adverse GI effects (stomach pain, nausea, vomiting, eructation, heartburn, indigestion) and lower adverse GI effects (bowel habit changes, diarrhoea, constipation, stool discolouration) with the prior oral iron product were documented. In addition, a record was made of intolerance with the prior oral iron product was made using the Gastrointestinal Symptom Rating Scale (GSRS, details in Reference [14]). Subjects completed the following questionnaires: baseline GSRS reflecting GI symptoms at least one week after stopping the prior oral iron product, Short Form-36 (SF-36) health related quality of life (https://www.rand.org/health-care/surveys_tools/mos/36-item-short-form.html) and a menstrual period questionnaire (details in Supplemental File) including the date, duration and blood flow of their last menstrual period.

Subjects were randomized into one of three treatment groups, but were blinded as to which group they were in using double-blind, double-dummy masking. Subjects were supplied with a six-week supply of study product and instructions of dosing. Subjects were instructed to follow their habitual diet and exercise routine and to not consume any disallowed medications/supplements (e.g. supplements that can chelate iron) that could interfere with the assessment of the study product for the duration of the study. Subjects were provided with an appointment to return to the study site at week 6.

At week 3 and 9, during phone visits, all subjects were asked to complete the GSRS questionnaire, and the menstrual period questionnaire, to record the date, duration and blood flow of their last menstrual period. They also were queried about adverse events and relatedness with IWP (unrelated, possibly-related, probably-related). Subjects returned to the clinic site at week 6 and 12. At each clinic visit, any changes in health status, medications and any adverse events, including relatedness with IWP, were documented. A fasting blood sample was collected and a full blood count, including ferritin, serum iron, transferrin saturation was measured. Subjects completed the following questionnaires: GSRS, the Short Form-36 (SF-36) and the date, duration and blood flow of their last menstrual period using the Menstrual Period Questionnaire. Subjects returned any unused study product and additional product was dispensed at clinic visit 3 (week 6). The adherence based on pill-count was determined at weeks 6 and 12. Subjects were instructed to continue following their habitual diet and exercise routine and to not consume any disallowed medications/supplements that could interfere with the assessment of the study product for the duration of the study.

**Sample Size and Statistical Analyses**

We require a minimum of 11 women per group on the assumption that the adherence rate of a population of women with a self-reported history of intolerance to oral iron will double from 40% to 80%, with a two-sided type I error rate of 5% (α =0.05) and power, 1-β = 0.80. This is based on a pilot survey amongst Active Iron users with a self-reported history of intolerance to iron. Only 37% were continuously adherent to oral iron therapy and 87% of these women reported themselves adherent with Active Iron 14mg. This sample size calculation assumes that the tolerability of higher doses will be similar to Active Iron 14mg and at least 11 women overall and in each dose group, regardless of the presence of anaemia, will be required to evaluate the primary objective. An interim evaluation when at least 15 women have completed will be used to confirm this. In addition, each arm is powered on the basis of the expected within group change in haemoglobin using following assumptions: an increase of 1.0 g/dL, standard deviation (σ) = 1.0, a two-sided type I error rate of 5% (α =0.05) and power, 1-β = 0.80. This is based on pilot observational evaluation of five women with mild iron deficiency anaemia at baseline (two pregnant) taking 14mg of study product daily for 12 weeks, the haemoglobin increased from 11.4 ± 0.9 g/dL at baseline to 12.5 ± 0.7 g/dL. Accordingly, we require in a minimum number of 8 subjects with anaemia overall and per arm. Accounting for potential drop-out, 10 women per arm will be adequate for this endpoint. Pre-specified subsets (e.g. heavy menstrual bleeding, blood donation) will be assessed unadjusted and with adjustment of outcome for baseline characteristics.

Descriptive data are presented as n (%) as well as either mean ± SD or median (25th:75th percentile) for normally and non-normally distributed continuous variables, respectively. Shapiro-Wilk’s test used to formally assess normality of the variable data. Frequencies and percentages (in parentheses) summarize categorical variables. If continuous variables were transformable to normal, they were power-transformed and independent, two sample t-tests were used for analysis of continuous variables. If data were not transformable to normal, non-parametric t-test equivalents (Wilcoxon signed rank and rank sum test, Mann-Whitney test and analysis of covariance [ANCOVA]) were used. Chi-squared (or Fisher Exact) analyses were used to compare categorical variables as appropriate. Repeated marker changes from baseline to 12 weeks (ferritin, haemoglobin, transferrin saturation) were also analysed using ANOVA with repeated measures models. Primary and secondary outcome measures were performed both with and without adjustment for the effects of baseline age, body mass index and systolic blood pressure. Further models included adjustment for pre-specified baseline outcomes of interest. Categorical endpoints were analysed using generalized linear modelling with a binomial outcome distribution for prevalence. A P-value of ˂ 0.05 was considered statistically significant.

**Additional Data**

**Table S1**. Prior iron products and dose groupings (daily elemental iron) taken by 57 of the 59 participants in the prospective study of the iron-whey-protein formulation. The proportion of participants in each dose grouping who reported stopping the prior iron product due to adverse GI effects is shown. Two additional participants did not know the iron product, one of which reported taking high dose iron. They did not bring the prior iron product to the baseline visit and both reported discontinuation of the iron product due to adverse GI effects.

|  | **Low dose group,**  **<14 mg** | **Medium dose group, 14-65 mg** | **High dose group,**  **>65 mg** |
| --- | --- | --- | --- |
| **Ferrous Sulfate** | 6 | 1 | 5 |
| **Ferrous Fumarate** |  | 3 | 35 |
| **Ferrous Gluconate** | 3 |  |  |
| **Ferrous Bisglycinate** | 1 | 1 |  |
| **Ferric Phosphate** |  | 1 |  |
| **Ferric Hydroxide** |  | 1 |  |
| **Total** | **10** | **7** | **40** |
| **Discontinued due to adverse GI effects, n (%)** | **8 (80)** | **5 (71)** | **33 (83)** |

**Table S2**. Multivariable linear regression model on changes in haemoglobin over the 12 week study period

|  | Estimate | Std. Error | t value | Pr(>\|t\|) | OR | 2.5 % | 97.5 % |
| --- | --- | --- | --- | --- | --- | --- | --- |
| Dose group | 0.015651 | 0.008723 | 1.794 | 0.0817. | 1.016 | 0.998 | 1.034 |
| Age | -0.017922 | 0.013549 | -1.323 | 0.1948 | 0.982 | 0.966 | 1.010 |
| Baseline Hb | -0.637077 | 0.120009 | -5.309 | 6.83e-06 *** | 0.529 | 0.414 | 0.675 |
| Baseline BMI | 0.052517 | 0.029795 | 1.763 | 0.0869. | 1.054 | 0.992 | 1.120 |
| Baseline SBP | 0.019714 | -0.011606 | -1.699 | 0.0985. | 0.980 | 0.958 | 1.004 |

Signif. codes: 0 ‘***’ <0.001 ‘**’ <0.01 ‘*’ <0.05 ‘.’ <0.1. Residuals: Min = -1.3969 ; 1Q = -0.5860; Median = -0.1319; 3Q = 0.5114; Max = 1.4576. Multiple R-squared: 0.5126, Adjusted R-squared: 0.4409, F-statistic: 7.151 on 5 and 34 DF, P-value: 0.00012.

**Figure S1**. Median adherence (medication-possession-ratio) per dose group taking IWP over the study period. There was no difference between the three dose groups; 95.0% (81.6, 100.4) 14 mg daily dose group; 100.6%(96.1, 103.0) 25mg daily dose group; 95.8% (82.9, 98.5) 50mg daily dose group, (Kruskal-Wallis test, P=0.077). Abbreviation: IWP= iron-whey-protein formulation.

A B

**Figure S2**. The overall Gastrointestinal Symptom Rating Score (GSRS) for other (previous) oral iron product (black circle) and for IWP over the course of the study (A, all P<0.001 versus previous oral iron product) and (B) the average GSRS data by dose group (ANOVA P=0.79) amongst 59 women with a history of intolerance to oral iron. An GSRS gut symptom score of 15 is a perfect GSRS score reflecting no adverse GI symptoms. Overall, women were 4.0 (95%CI 2.3 to 7.0, P<0.0001) more likely to experience constipation and 3.2 (95%CI 1.7 to 6.2, P=0.0002) more likely to experience abdominal pain with the prior oral iron product than with IWP.

Abbreviations: IWP= iron-whey-protein formulation; GSRS=Gastrointestinal Symptom Rating Scale gut symptoms score.

**Additional Details on Elicited Adverse Gastrointestinal Effects and Gastrointestinal Symptom Rating Scale**

Only one woman (4.3%) reported adverse GI effects (constipation, dark stools and excess flatulence) that were probably related to IWP. In this case, the woman was taking IWP 14mg, all symptoms were considered mild and the woman was happy to persist with treatment for 12 weeks. Coupled with the 5 people who withdrew from the study, at least 53 (89.9%) women taking IWP were free of adverse GI effects and women were at least 9.8 (95%CI 4.6 to 21.0, P<0.0001) times more likely to report adverse GI effects that were probably related to oral iron with the previous oral iron product. In addition, 22 participants reported side effects that were “possibly” related to IWP. Most of these (n=17, 77.3%) reported mild symptoms, 3 (13.6%) reported moderate symptoms and 2 women (9.1%) reported severe symptoms. Overall, using reliable change index with 95% confidence,[21] 45 (81.8%) women had an improvement in GSRS using IWP compared to the previous iron product. Using reliable change index with 95% confidence, more women who previously took higher dose oral iron (>60mg elemental iron, n=35, 83.3%) had improved gut-symptom-scores on IWP compared versus those taking lower dose oral iron previously (n=10, 58.8%, P=0.045). Accounting for three further women who withdrew due to adverse GI effects and one woman lost to follow up, these data show 4.25 (95%CI 2.15 to 8.39, P<0.0001) more women had adverse GI effects when taking the previous oral iron product.

**Figure S3**. Detailed SF-36 data at baseline broken down according to the 8-health related quality of life concepts: physical functioning, bodily pain, role limitations due to physical health problems, role limitations due to personal or emotional problems, emotional well-being, social functioning, energy/fatigue, and general health perceptions. Using ANOVA, there were significant differences across the domains (P<0.0001) and SF-36 Energy/Fatigue domain scores in this population (60.1 $\pm$3.4%) were significantly impaired compared to all the other domain scores at baseline (all P<0.001).

**Figure S4** Changes in mean SF-36 Energy/Fatigue from baseline to 12 weeks in each IWP dose group in women with iron deficiency anaemia with a history of intolerance to oral iron. SF-36 Energy/Fatigue scores increased overall from 60.9 ± 3.4% to 71.2 ± 2.6 % (T-Test, P=0.0007). Abbreviations: IWP= iron-whey-protein formulation; SF-36 = short form 36 health related quality of life score.

**Menstrual Period Questionnaire**

**MENSTRUAL PERIOD QUESTIONNAIRE**

Do you suffer from heavy periods?

☐ Yes ☐ No

How long does your menstrual period usually last?

☐ less than 3 days

☐ 3 to 7 days
☐ more than 8 days

Do you experience menstrual flow that soaks through one or more pads or tampons every hour?

☐ Yes ☐ No

Do you need to use double sanitary protection to control your menstrual flow?

☐ Yes ☐ No

Do you experience menstrual flow that includes large blood clots?

☐ Yes ☐ No

Does your menstrual flow inferfere with your regular lifestyle?

☐ Yes ☐ No

Do you experience pain during your period?

☐ Yes ☐ No

Do you have other symptoms like easy bruising?

☐ Yes ☐ No

Do you take medications during your period?

☐ Yes ☐ No

If yes, please give details _______________________________________________________________________________________________________________________________________________________________________________________________________________________________________________________


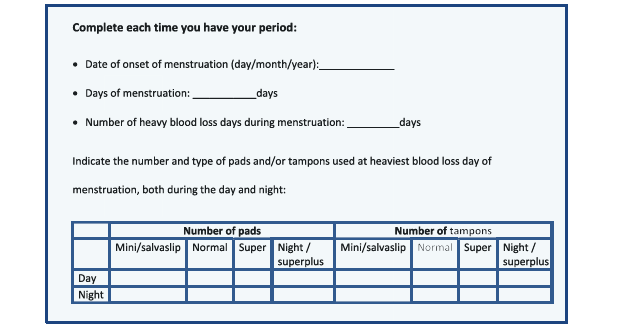

Supplement: Supplementary file 1 — Supplementary file1 (DOCX 231 KB) [file 11096_2023_1640_MOESM1_ESM.docx]
